# Implementing Activity-Based Therapy for Spinal Cord Injury Rehabilitation in Canada: Challenges and Proposed Solutions

**DOI:** 10.3390/healthcare12070703

**Published:** 2024-03-22

**Authors:** Hope Jervis-Rademeyer, Lovisa Cheung, Nicole Cesca, Cindy Gauthier, Kristen Walden, Kristin E. Musselman

**Affiliations:** 1 Department of Medicine, Faculty of Medicine and Dentistry, University of Alberta, Edmonton, AB T6G 2R3, Canada; 2 Rehabilitation Sciences Institute, Temerty Faculty of Medicine, University of Toronto, Toronto, ON M5G 1V7, Canada; lovisa.cheung@utoronto.ca (L.C.); nicole.cesca@mail.utoronto.ca (N.C.); kristin.musselman@utoronto.ca (K.E.M.); 3 KITE Research Institute, Toronto Rehabilitation Institute, University Health Network, Toronto, ON M4G 3V9, Canada; 4 Department of Physical Therapy, Temerty Faculty of Medicine, University of Toronto, Toronto, ON M5G 1V7, Canada; 5 School of Rehabilitation, Faculty of Medicine, University of Montreal, Montreal, QC H3N 1X7, Canada; 6 Praxis Spinal Cord Institute, Vancouver, BC V5Z 1M9, Canada

**Keywords:** exercise therapy, neurological rehabilitation, spinal cord injury, qualitative methods

## Abstract

Activity-based therapy (ABT) is a therapeutic approach with multiple benefits including promoting neurorecovery and reducing the likelihood of secondary complications in people living with spinal cord injury (SCI). Barriers and facilitators to ABT implementation for SCI rehabilitation have been studied from various perspectives through qualitative research. However, these viewpoints have not been synthesized to identify challenges of and strategies for implementing ABT across the Canadian healthcare system. Thus, the purpose of our study was to examine the current state of ABT in Canadian healthcare settings according to users’ perspectives. Our main objectives were to compare barriers and facilitators to ABT implementation across Canadian healthcare settings according to users’ perspectives and to identify optimal intervention strategies for ABT delivery across the Canadian healthcare system from acute to community care. We searched Scopus, CINAHL, OvidMedline, and other sources. Eligible articles were qualitative or mixed methods studies exploring ABT for adults with SCI in a Canadian healthcare setting. We analyzed qualitative findings through a thematic synthesis followed by a deductive content analysis. The Mixed Methods Appraisal Tool was used for critical appraisal. Nine articles were included. The thematic synthesis revealed two main themes: (1) factors influencing acceptance and adaptation of ABT across healthcare settings in Canada and (2) proposed solutions. The deductive analysis applied the Behaviour Change Wheel (BCW) to identify limited components of behaviour and appropriate interventions. To address ABT implementation challenges across the Canadian healthcare system, evidence-based interventions should target BCW subcategories of reflective motivation, social opportunity, and physical opportunity.

## 1. Introduction

Spinal cord injuries (SCIs) are life-altering events that result in chronic impairments affecting sensory, motor, and autonomic function below the level of injury [1]. These impairments can greatly affect the quality of life of people living with an SCI. Indeed, in addition to their initial impairments, these individuals have more risks of developing secondary health complications, such as cardiovascular dysfunction or musculoskeletal impairments [2], and decreased mobility and independence.

Recent years have witnessed a significant shift in SCI rehabilitation in Canada, going from a compensatory to a more restorative approach. With the aim of maximizing recovery, independence, and quality of life, activity-based therapy (ABT) has emerged in different rehabilitation settings. ABT is a therapeutic approach with multiple benefits including promoting neurorecovery and reducing the likelihood of secondary complications in people living with SCI. Indeed, ABT interventions are based on neuroplasticity principles and involve task-specific neuromuscular activation below the level of injury with a high dosage (e.g., many repetitions, increased exercise time) and a moderate–high cardiorespiratory intensity [3,4].

ABT is often associated with the use of technology such as electrical stimulation devices, since these pieces of equipment help deliver high doses of training and stimulation [5,6,7]. However, ABT interventions are not limited to their use [8]. Healthcare facilities have reported using manual stimulation and conventional gym equipment to provide ABT interventions [9]. Numerous creative techniques can be used with low-technology equipment as long as ABT principles are applied [4].

ABT has been widely recognized and embraced by individuals with SCI as a significant component of their rehabilitation and their SCI journey [10]. They attribute part of their improvements, achievements, and well-being to their participation in ABT activities [10]. Despite the numerous documented health and well-being benefits, importance for people with SCI, and the fact that it requires no specialized equipment, ABT implementation and accessibility are still limited across the Canadian continuum of care [10,11].

The ABT Community of Practice (ABT CoP) is a national collaborative group of individuals living with SCI, clinicians, researchers, administrators, and other groups who have an interest in ABT [12,13]. The main goal of this group is to improve access to and quality of ABT for people living with SCI [12]. Over the past few years, several qualitative research studies have been conducted by members of this group to better understand the current use of ABT [9,10,14,15]. The perspectives of different stakeholders across the continuum of care from acute care to the community centers have been captured. Each stakeholder reported experiencing multiple barriers and facilitators to ABT implementation, and elements limiting ABT implementation were identified at various levels from individual factors to environmental and organizational factors [9,10,11,14,15].

Although we have a better understanding of the barriers and facilitators in different settings across the Canadian healthcare system [9,10,11], these viewpoints have yet to be synthesized to identify common challenges and strategies for implementing ABT. Thus, our study aimed to examine the current state of ABT by comparing the barriers and facilitators to ABT implementation across Canadian healthcare settings according to users’ perspectives. We then used the Behaviour Change Wheel (BCW) to identify optimal intervention strategies for ABT delivery across the continuum of care from acute to community care settings.

## 2. Methods

### 2.1. Study Design

A qualitative thematic synthesis, as outlined by Thomas and Harden, was conducted [16]. This methodology was chosen for its capacity to integrate findings from a variety of qualitative studies while ensuring that the authenticity of the original research results was preserved [16].

This review adhered to the enhancing transparency in reporting the synthesis of qualitative research (ENTREQ) guidelines to ensure a thorough and methodological approach [17]. The application of this guideline reinforced methodological rigour in the reporting process [17].

### 2.2. Article Identification

A systematic approach was used to identify articles, with a focus on evaluating the relevant population, concept, and context. Adults with SCI were the target population, ABT was the concept of interest, and the context was defined as the Canadian healthcare system. In December 2023, a comprehensive, pre-planned search was conducted across three databases including Scopus, CINAHL, and Ovid MEDLINE. A review of other sources such as reference lists of relevant articles was also completed to ensure that no articles were missed. The search strategies were collaboratively developed by researchers and drew upon the search strategies from prior reviews [18]. An example search strategy used in one of the databases is provided in Appendix A.

Following the initial retrieval of articles, duplicates were removed, and the remaining articles underwent title and abstract screening. Title and abstract screening and subsequent full-text reviews were conducted by two researchers (N.C. and K.E.M.). Any disagreements in the selection process were resolved through discussion, and a third researcher (K.W.) confirmed the final selection of articles.

**Figure 1:**
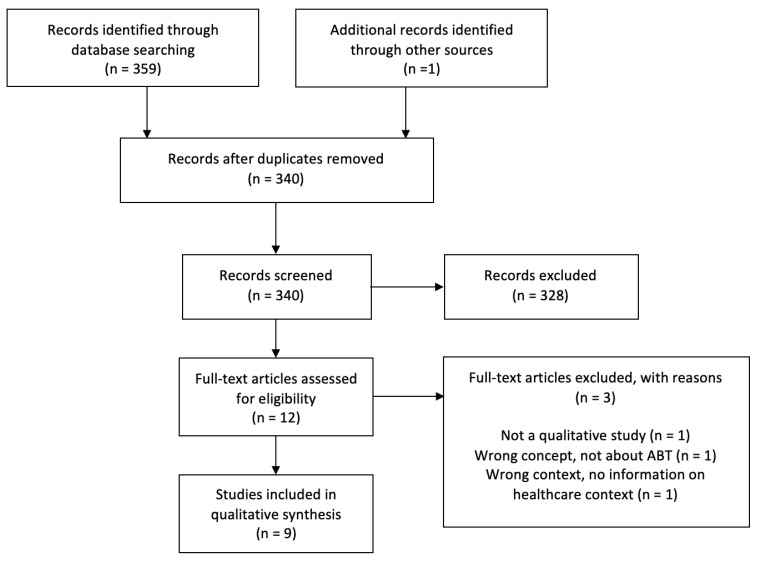
PRISMA flow diagram of included studies.

Articles were included if they (a) used a qualitative or mixed methods study design; (b) explored the perspectives of individuals knowledgeable about SCI and ABT (e.g., clinicians, therapists, clinic administrators, individuals living with SCI); and (c) were conducted within the Canadian healthcare system. Articles were excluded if they focused solely on neurological populations other than SCI, investigated physical activity or other therapies that do not fall under the definition of ABT [19], or were conducted outside the Canadian healthcare system context (e.g., laboratory settings). All quantitative studies, conference proceedings and abstracts, textbook chapters, and reviews were also excluded. Figure 1 contains the PRISMA flow diagram detailing the article selection process.

### 2.3. Data Extraction and Critical Appraisal

Data extraction was completed by two researchers (N.C. and L.C.). Study details such as the primary author, year of publication, study methodology and objectives, inclusion criteria, healthcare setting, population characteristics, sex/gender, number of interviews/focus groups, and major findings were extracted by N.C. L.C. extracted data categorized as “Results” as well as associated quote tables from each included study. A critical appraisal of each included study was conducted independently by two researchers (H.J.-R. and L.C.) using the Mixed Methods Appraisal Tool (MMAT). Details of this appraisal are outlined in the Supplementary Materials. This tool is designed for the critical evaluation of studies with diverse methodological designs within systematic reviews. Each study design category comprises two screening criteria along with five criteria to assess methodological quality. As the MMAT advises against calculating an overall quality score for each article, a comprehensive breakdown of the ratings for each criterion was employed instead [20].

### 2.4. Qualitative Thematic Synthesis

The analysis of the included articles adhered to the structured three-step approach to qualitative thematic synthesis as outlined by Thomas and Harden, line-by-line coding, development of descriptive themes, and generation of analytical themes [16], and was modelled after a previously completed synthesis [21]. First, an inductive approach was employed where two researchers (L.C. and H.J.-R.) independently coded the results from a single article and subsequently convened to compare and discuss their codes and create a preliminary codebook. This collaborative approach was used to maintain consistency and rigour in the analysis. Following the initial coding, L.C. and H.J.-R. proceeded to code the results of the remaining eight articles separately, with each researcher independently coding four and adding new codes to the preliminary codebook. Next, a meeting was held to discuss line-by-line coding (Step 1) and to collaboratively develop descriptive themes, sub-themes, and categories (Step 2), after which a refined codebook was created. The third step (Step 3) involved a collaborative discussion with a third researcher (C.G.), which focused on generating analytic themes and confirming the final codebook. During these discussions, themes, sub-themes, and categories were critically evaluated and finalized, ensuring a comprehensive and accurate representation of the data. This iterative process allowed for a dynamic refinement of themes, sub-themes, and categories, ensuring that they accurately represented the data.

Following the inductive thematic synthesis, L.C. and H.J.-R. applied the findings to subcomponents within the Capability Opportunity Motivation—Behaviour (COM-B) model of behaviour change [22]. Specifically, the challenges to ABT implementation identified through the thematic synthesis were mapped onto Michie et al.’s BCW [22]. The BCW was developed by drawing on 19 frameworks designed to categorize behaviour change interventions. At the centre of the Wheel is the target behaviour, which in the current study is the implementation of ABT across the Canadian healthcare continuum. Moving outward, the first BCW ring consists of the sources of behaviour (i.e., COM-B components). The challenges to ABT implementation were deductively classified into one of the six key subcomponents: (1) physical capability, (2) psychological capability, (3) social opportunity, (4) physical opportunity, (5) reflective motivation, and (6) automatic motivation. This analysis was executed through a classical (deductive) content analysis approach. The COM-B model, a framework for understanding behavioural change [22], provided a structured lens through which the data could be viewed and interpreted. A subsequent meeting with C.G. occurred to finalize this deductive analysis. This step was essential for ensuring that the application of the themes, sub-themes, and categories to the COM-B model subcomponents was thorough and accurately reflected the data.

Next, using the BCW, L.C. linked the components of the COM-B to their corresponding intervention functions [22]. This alignment was critical for identifying potential intervention strategies that could be derived from the synthesized data. Within the BCW, each source of behaviour (i.e., COM-B component) includes intervention functions (e.g., modelling, coercion). For instance, challenges falling within physical opportunity would be addressed using intervention functions including training and environmental restructuring [22]. Furthermore, at least one behaviour change technique (BCT) is part of each intervention function [23]. A BCT is an active part of the intervention function that is specifically designed to affect behaviour. To give one broad example, demonstration of the behaviour (BCT) may be selected as part of training (intervention function) which affects automatic motivation (source of behaviour) to improve the target behaviour.

Throughout this synthesis process, a balance was maintained between inductive and deductive approaches, ensuring both a grounded understanding of the data and their application to the established behavioural change theory. This comprehensive approach allowed for a nuanced and in-depth thematic synthesis, contributing valuable insights to the field of study.

## 3. Results

### 3.1. Included Studies

The database search yielded a total of 340 articles after de-duplication. After title and abstract screening of these articles, 12 were retained for full-text review. Nine articles met the study’s inclusion criteria [9,10,11,14,15,24,25,26,27].

A description of the included studies is detailed in Table 1. With respect to methodology, eight of the included studies were of qualitative design, while one study used a mixed methods approach. Eight studies focused on ABT for people with SCI, and one study included perspectives from a mixed neurological population (Table 1). Of 131 total participants, the perspectives of 121 participants from eight key interest groups were included: physical or occupational therapists (n = 47), people living with SCI (n = 34), kinesiologists (n = 6), community exercise trainers (n = 12), clinic directors (n = 2), hospital or community administrators (n = 8), researchers (n = 7), and advocates, funders, and policy experts (n = 5). Participants were from various provinces across Canada, including Ontario, Alberta, Quebec, Saskatchewan, and Nova Scotia. Regarding participant sex, seven studies comprised a mix of male and female participants, while two articles did not report the sex of participants [15,24]. Studies included perspectives from acute care, rehabilitation, community, and non-SCI-specialized centers (Table 1).

### 3.2. Context

Participants described ABT as an approach that was diverse and client-centered [9,10,11,14,25,26,27]. Due to the broad range of client goals and clinician backgrounds, ABT was delivered in creative ways using various equipment, technologies, and hands-on techniques [9]. The critical principles of ABT that were used in therapy included working below the level of injury [9,10,11], incorporating “*very high repetition*” [9] and high intensity movement, focusing on “*task-specific movement*” [11], involving “*loading, weight bearing*” [9] movements such as locomotor training [26,27], and incorporating sensory stimulation such as functional electrical stimulation [9,11,14]. ABT was reported by clinicians to be “*for everyone*” [9] in the SCI population, including those at different stages of recovery, levels and severities of injury, and ages. Participants with SCI described positive experiences and benefits as a result of participation in ABT, including “neurological recovery” [10], “reducing the occurrence of secondary complications associated with SCI” [10], “positive impact on their mood and decreased symptoms of depression” [26], “transferrable gains” [27] leading to improved function in the community, and “social connection” [25].

### 3.3. Qualitative Thematic Synthesis

The synthesis of the results of the included articles revealed two main themes: Theme 1: factors influencing acceptance and adaptation of ABT across healthcare settings in Canada, and Theme 2: proposed solutions. Table 2 provides an outline of the themes, sub-themes, and categories developed from the thematic synthesis. Additional supporting quotations are available in the Supplementary Materials. Italicized quotations represent speech spoken by the participants of the included studies, whereas non-italicized quotations refer to text written by the authors of the included studies.

Theme 1: Factors influencing acceptance and adaptation of ABT across healthcare settings in Canada.

This theme refers to the factors that enabled or limited the implementation of ABT across the Canadian healthcare system. Three sub-themes were captured within this theme: (a) identity, (b) knowledge, and (c) health system. Each of these sub-themes comprised several categories (Table 2). It is important to note that while ideas were categorized into distinct categories, some overlap may exist.

#### 3.3.1. Identity

*Person-specific considerations.* Various patient-related factors were considered by clinicians and people with SCI in the context of ABT implementation or participation [10,11,14,15,26]. Clinicians working in acute care and rehabilitation hospitals emphasized that patient activity tolerance and medical complexity influenced the extent of their ABT approach [14,15]. For instance, an acute care occupational therapist explained how some people with SCI “could not meet the dosage required for ABT due to a lack of tolerance” [14]: “*In the acute phase it’s so exhausting for them to do 20–30 min, right? It’s just exhausting for them*” [14]. It was also explained that therapists had “*to check with the medical team if [ABT] is appropriate*” to ensure that their patients were medically cleared to participate [14]. In rehabilitation hospitals, “perceived patient tolerance to ABT was a key factor in therapists’ delivery of ABT” as well [15]. Clinicians working in rehabilitation also “expressed a hesitancy to engage patients in ABT” when they perceived their patients to be experiencing a “mourning period, where patients were getting used to the diagnosis of SCI and what that meant to them” [15]. People living with SCI also reported individual-level challenges that influenced the extent of their ABT participation: “*I was doing full sit-ups and I ended up shearing my tailbone*” [10], “*when you’re not able to do a movement, and then it gets frustrating*” [10], and “*my body wasn’t used to that…And the hardest part with me was in the beginning, because I didn’t have the endurance*” [26]. Finally, input from hospital administrators corroborated these findings: “hospital administrators were concerned that individuals with SCI were not physically capable or emotionally ready to participate in intensive therapy early postinjury” [11].

*Professional roles and dynamics.* Roles and dynamics within the professional environment were found to influence decisions surrounding the application of ABT [9,11,15]. For instance, a community occupational therapist stated, “*just with my training personally […] as an OT, we’re not doing as much of that*” [9]. However, despite differences in professional training, it was “voiced strongly by hospital therapists, community trainers, hospital and community administrators, and persons with SCI” that various support staff, trainees, and volunteers were necessary to deliver ABT [11], signifying the importance of a large, multi-disciplinary team. Moreover, while some clinicians assumed roles such as “advocates” for approaches like functional electrical stimulation or “liaisons” between healthcare settings, other clinicians “resisted becoming a learner with equipment with complex technology” [15]. A physical therapist working in a rehabilitation hospital explained the reluctance to deviate from current routines or equipment:

*“It’s also specifically with just a higher-level technology. The initial learning curve is varied. Like a lot of therapists will approach it and say, “If this isn’t better or faster than what I’m currently doing, why should I bother with it?” It’s hard for them to get comfortable enough to make it faster than the standard therapy because they’re not using it”*.

[15]

At times, the structure of the therapy program also influenced the ability of different healthcare professionals to work together: “*Because our groups are run specifically by an OT assistant or a physio assistant, we can’t cross refer*” or “*We talk as a team before making a decision*” [15]. Moreover, some departments had to compete for limited resources, such as grants and equipment, which “contributed to a disconnect between departments” [15].

*Goals of therapy.* Competing goals in therapy influenced the implementation of ABT [9,10,11,14,15,24,25,26,27]. In acute care, people with SCI had “to be stabilized before engaging in therapy”, and thus safety was prioritized [14]. In the rehabilitation setting, clinicians typically set goals aimed at optimizing functional tasks; however, as stated by a physical therapist, “*you have a lot of competing goals to address*” [15]. Hospital clinicians often had to prioritize discharge, which “could lead to a compensatory approach that was at odds with ABT’s focus on neuro-recovery” [15]. People with SCI also had many of their own personal goals, such as improving walking, upper limb function, strength, endurance, or independence [9,10,24,25,26,27]. The breadth of these goals led to the need for clinicians to “individualiz[e] exercise prescriptions” [25], develop “*personal treatment plans*” [9], and “*get creative*” [9]. However, tracking ABT to determine progress towards meeting these goals has proven difficult [11]. Specifically, “some improvements individuals make don’t get captured on any tools they use” due to lack of sensitivity [11]. Moreover, researchers, advocates, funders, and policy experts stated that there is “no standardized approach to the collection of outcomes” and that “validated tools to measure function […] were not being used consistently by clinicians” [11].

#### 3.3.2. Knowledge

*Setting-specific considerations.* Several challenges specific to the healthcare setting were noted by clinicians, ultimately making it difficult to meet the intensity associated with ABT [14,15,24]. These challenges were reflective of clinicians’ knowledge about ABT. For instance, physical and occupational therapists working in non-SCI-specialized centres in Canada reported that they treated people with SCI in their facilities “because of limited vacancy at specialized centers” [24]. Moreover, in line with the increasing number of non-traumatic SCI cases in Canada, people with non-traumatic SCI were “more commonly being referred to their non-specialized center instead of SCI-specialized facilities” [24]. Working with people with SCI in non-SCI-specialized centres came with unique challenges pertaining to “gaps in specialized knowledge related to SCI and ABT” (e.g., lack of foundational knowledge about working with people with SCI, lack of awareness of ABT), “lack of access to resources for SCI rehabilitation and ABT implementation” (e.g., lack of equipment needed to work with those with SCI, lack of community resources), and “limited time for therapy and ABT delivery” [24]. Overall, it was found that clinicians working in non-SCI-specialized centres were particularly lacking in knowledge about ABT [24]. On the other hand, clinicians working in SCI-specialized acute care settings were knowledgeable about the high intensity associated with ABT but explained that medical acuity of people with SCI disrupted opportunities to engage in therapy:

*“From a respiratory point of view, they crash, then that’s when they’ll go down to the ICU and then they’ll come back up again and when patients have a big crash like that then you’re resetting the clock every time because they’ve got to start again when they come back up to us”.*

A challenge specific to SCI-specialized acute and rehabilitation centres was the emphasis on discharge, as previously discussed [14,15]. Clinicians demonstrated knowledge that ABT was a remediative approach targeting below the level of injury in people with SCI; however, discharge timelines introduced challenges. In acute care, a person “*without any other sort of trauma [*…*] a very clean injury*” [14] would have a short length of stay and then be discharged, with little time to introduce ABT into their therapy program. In rehabilitation, preparing a patient quickly for discharge often meant favouring a compensatory over remediative approach to complete activities such as “dressing, toileting, transferring” [15].

*Current understanding of ABT definition and principles.* We identified a lack of knowledge and understanding about ABT across multiple key interest groups and across healthcare settings [9,10,11,14,15,24]. Much of the uncertainty stemmed around a lack of clarity on the definition of ABT, “what it encompassed” [11], and the key principles that were associated with ABT. While some people with SCI, community administrators, and trainers were familiar with key characteristics of ABT, several “hospital therapists pointed out that once they learned about the definition of ABT they recognized that they actually practiced ABT to a much greater extent than they realized” [11]; this realization was also mirrored by therapists in non-SCI-specialized centers [24]. In acute care, “most sites incorporated some form of ABT into therapy sessions but were hesitant to say that it met the definition provided” and were “unaware of the research evidence validating ABT for individuals with SCI” [14]. Rehabilitation clinicians were familiar with some but not all principles of ABT; for instance, an occupational therapist “did not know about providing stimulation below the spinal lesion” [15]. In the community, clinicians explained how the lack of knowledge about ABT stemmed from the “*mainstream medical mindset […] that there is no value in working below level of injury*” [9]. This led to a “lack of knowledge amongst healthcare professionals” and clients: “*they just don’t know about us; they don’t know about activity-based training because they’re not taught it in the hospital setting*” [9]. People with SCI also indicated “no referral through healthcare professionals in early rehabilitation, and that they heard about ABT from a friend or through the media” [10] and needing to teach clinicians themselves: *“I was always now, teaching them how to do ABT and explaining the philosophy of it*” [10]. Overall, clinicians and other key interest groups reported a lack of quality ABT education and training across healthcare settings and expressed a desire to increase knowledge about ABT [9,11,14,15,24]. We also found that evidence was lacking with respect to the optimal timing, dosage, and intensity of ABT, which would help inform the development of standardized ABT guidelines [11].

#### 3.3.3. Health System

*Cost.* The challenge of funding was evident across the continuum of care and across provinces [9,10,11,15,27]. Clinicians working in rehabilitation hospitals stated that certain equipment was “not necessarily covered by departmental funding” [15], and equipment funded through research grants also had limitations applied to its use [15]. Moreover, medical equipment was often perceived as difficult to import to Canada due to “accompanying tariffs and duties” [15]. The high cost of “shipping, purchasing, and/or maintaining equipment” [9] was also voiced by community-based clinicians, such as a kinesiologist who stated, “*it’s quite expensive to up-keep the machines*” [9]. Researchers even stated that “high-tech, costly robotics like the Lokomat […] has not demonstrated superior benefits to justify their costs” [11]. Clinicians working in the community also perceived the cost of ABT to be “*the biggest barrier*” [9] for their clients. A kinesiologist explained the implications of high dosages and intensity of ABT on cost: *“the more that you exercise, the better you feel and the more results you see, but you have to pay every time you go to the gym*” [9]. Given that community-based clinics were not publicly funded, clients either had to “pay completely out of pocket” or use insurance coverage if the individual was “*injured in a car or at work*” [9]. Unfortunately, however, there was often a lack of coverage provided by third parties [11]. People with SCI also reported the cost of ABT as “*one of the biggest limitations*” [10]*,* “*especially if you want to go on a consistent basis*” [10,11]. To combat these challenges, individuals with SCI had to turn to fundraising to cover ABT costs [10], while community clinics had to apply for charity status to “receive donations, host fundraising events, apply for grants, and maintain affordability for clients” [11]. Some people with SCI even resorted to exercising at home or at a regular gym, as this arrangement was more “financially feasible than long-term participation in an intensive community rehabilitation program” [27]. Researchers, advocates, funders, and policy experts stressed the need to perform “economic evaluations to determine the cost-benefit analysis, attract government funding, and support implementation of ABT in various settings” [11].

*Equipment, technology, and facilities.* Clinicians experienced difficulties using or accessing equipment or technology in their facility [9,11,14,15,24]. In rehabilitation hospitals, clinicians had to share “high-tech equipment” [15] between departments to be more cost-effective; however, this arrangement introduced difficulties of storing equipment in other departments and “competing for scheduled time” [15]. In the community setting, clinicians described needing to be selective with their equipment due to a lack of floor space in their facility: “*we only have so much physical floor space*” [9]. Several community-based clinicians also described some technologies, such as electrical stimulation, as “cumbersome” or “finicky” [9], which influenced their ABT approach when working with people with SCI. Although there were challenges to accessing or using certain types of equipment or technology, a kinesiologist emphasized that they were used “*as more a supplement than a crutch*” [9]. In non-SCI-specialized centers, clinicians reported not having the appropriate equipment to use to treat individuals with SCI: “*don’t always have all of the resources, especially like physical equipment*” [24]. Acute care clinicians reported using “small portable equipment, like a handheld electrical stimulation device” [14], but enhanced cleaning protocols in their setting made use more challenging during the COVID-19 pandemic. Moreover, clinicians, researchers, and administrators emphasized a lack of and need for “training on the appropriate use of equipment and adapting the way equipment is used maximize the potential benefits” [11].

*Travel and transportation.* Several studies discussed the challenge of commuting long distances for ABT, especially for people with SCI who have limited mobility options [9,10,24,26]. An occupational therapist working in the community explained the importance of making good use of their clients’ time: “*it’s a huge responsibility too, when they’re paying out of pocket and they’re coming to see you from an hour and a half away. You’re like, ‘well I better make some change’*” [9]. The far commutes were explained to be the result of a lack of facilities delivering ABT: a physical therapist stated, “*it also comes down to finances … and, I think you’re right, travel, being able to get to those locations cause there’s not a lot of them*” [9]. A person with SCI who had been participating in ABT for three years explained, “*there aren’t many options. Like for me, [ABT Facility] is the only facility within an hour’s drive of my house*” [10]. Another individual with SCI reported spending “up to five hours a day commuting” for ABT [26]. The desire for therapy to be close to home was a determining factor influencing the decision for people with SCI to attend a non-SCI-specialized centre instead of “*a spinal center outside the city [because they] wanted to come back home*” [24] or even “*starting up our own little kind of exercise-based facility here*” [10].

*Time.* Although people with SCI generally described ABT as a worthy time commitment [10,26,27], clinicians across all healthcare settings reported challenges with respect to time [9,11,14,15,24]. Acute care clinicians described having limited time to deliver repetitive movement due to heavy and unpredictable caseloads: an occupational therapist highlighted, “*[Caseload] can be unpredictable. You can come to work and you kind of know what your day is going to be like and then by 9:30 [am] you’re already off the tracks*” [14]. With respect to using technology, clinicians working in a rehabilitation hospital described having to consider “potential setup time required to use technology” [15] and that training on new technologies was “*time consuming and intensive*” [15]. Training in community clinics was also reported as lengthy: “*it does take a long time because we train everybody on the technology*” [9]. Despite this, a kinesiologist emphasized the importance of taking time to learn about new technologies: “*if people understand and know how to use it and what it does and its benefits, then they’re more likely to use it*” [9]. Many difficulties with time were expressed by clinicians working in non-SCI-specialized centres including having limited time to dedicate to each patient due to “high caseloads” and needing to use specialized equipment which was reported as “*very time-consuming*” [24]. A clinician working in a non-SCI-specialized centre explained the challenge of delivering high dosages of ABT to people with SCI: “*is very challenging to do, [as it’s] very dependent a lot on timing and that doesn’t exist*” [24]. This sentiment was also mirrored by researchers, hospital clinicians, and administrators [11], such as when working with “clean” clients with short lengths of stay in rehabilitation [15].

*Staffing.* Staffing limitations were also found to impact ABT implementation [9,14,15]. As stated by a physical therapist working in acute care, “*Staffing and patient flow tend to be the bigger elephants in the room, unfortunately*” [14]. To supplement therapist-led sessions, patients’ social supports helped deliver components of the ABT program to achieve the high dosages associated with ABT [14,15]. This challenge was also reflected in the rehabilitation setting: “Staffing requirements were often recognized as a perceived barrier because of the training and experience required” [15]. To alleviate these staffing demands, complementary therapies were offered, such as fitness and recreation [15]. In the community, staffing varied from preferentially employing one type of clinician to having a highly interprofessional team, “*all of our full-time staff are kinesiologists”* or “*we’re left and right hands […] not many clinics that have OT and PT in the same spot*”, and depended on factors such as demand and funding [9]. Although staffing challenges were evident across the continuum of care, people with SCI felt supported and motivated through the presence of staff and valued the social connection developed with peers and volunteers [25].

*Transitions in care.* Importantly, transitions across the continuum of care introduced challenges in maintaining the engagement of people with SCI with ABT [9,10,11,14,15,27]. An acute care physical therapist highlighted the disconnect with other settings after a patient was discharged: “*We can’t really speak to [ABT use in the community] because we don’t really follow up with them after they’ve been discharged from acute care*” [14]. Lack of continuity was especially apparent for clinicians working in mixed neurological units, whereas the continuity of ABT was easier in “specialized units” for SCI [14]. In rehabilitation, an occupational therapist explained attempting to minimize the disconnect between rehabilitation and the community by “*do[ing] site visits just to see kind of what [community ABT clinics] are doing, what they have to offer, and to better understand the tenets of their program*” [15]. However, people with SCI were sometimes discharged from inpatient rehabilitation just when they became physically or mentally ready to participate in ABT, and so “timing was misaligned” [11]. People with SCI who were discharged from an outpatient rehabilitation program reported disappointment and difficulty transitioning into the community due to the absence of guidance and structure [27]. Individuals with SCI described challenges finding “competent community rehabilitation programs” and experienced a “decline in their psychological well-being” [27]. One participant explained, “*After that program finished, when I came home in December, it was obviously the holidays and after that I got really depressed*” [27]. What eased the transition into the community were “home exercise programs and community resource pamphlets” [27]. Community clinicians explained how the disconnect across settings stemmed from how “*in Canada, activity-based training isn’t very well known and it doesn’t have a lot of back up from the medical community”*, as well as differences in goals across the continuum: *“public system is orientated towards discharge”* [9] and “*compensating*” [10].

Theme 2: Proposed solutions.

This theme consisted of two sub-themes with no additional categories: (a) motivation, empowerment, and advocacy for ABT and (b) desire for education and training (Table 2).

#### 3.3.4. Motivation, Empowerment, and Advocacy for ABT

Overall, the participants of included studies emphasized the importance of advocating for ABT across the healthcare system and motivating and empowering people with SCI to participate in ABT [9,10,11,25,26]. Specifically, people with SCI and clinicians expressed a desire to introduce “ABT earlier post-injury” [11]: “*I wish more people had access to it and more people in places like rehab knew more about it*” [10] and “*activity-based training should be implemented in the acute care setting*” [9]. It was explained how “increased and earlier implementation of ABT will lead to a more streamlined continuum of care and help “*instill in them [clients] the need for ongoing movement their whole life*” [9]. However, given “*the lack of awareness and lack of education*” [9] about ABT, there was ultimately a lack of support for ABT across the healthcare system. Moreover, not only do clinicians, policy makers, funders, and other key interest groups working across the healthcare sector need to increase their understanding of ABT, but people with SCI also need to be motivated and empowered to participate [10,25,26]. Participation in ABT can be positively influenced by the high intensity of ABT programs [26], by providing participants with realistic hope rather than false or no hope [10], exercising in a group setting [25], and incorporating educational components [26]. A participant with incomplete SCI explained the importance of group interactions in maintaining her engagement:

*“It’s really hard to motivate yourself when you’re by yourself living alone, and someone says, well, did you do your sit to stands today? Well no, you know? Because you’re just not motivated. And in a group like that, and yes we knew everybody by the time we ended, we could laugh and have a good time with it”.*

[25]

Moreover, another person with SCI described how having been “*explained the whole body from top to bottom*” had helped her “*[feel] more in control of the direction of your rehab going forward*” [26].

#### 3.3.5. Desire for Education and Training

A desire for ABT education and training opportunities was voiced by many participants across the included studies [9,10,11,14,15,24]. As expressed by a rehabilitation occupational therapist, “*we just don’t have specialized training in certain things*” [15], and a rehabilitation physical therapist, “*I think more investment in training and education. There are no exams crafted, or recognition, here. Those things like recognizing champions is nonexistent*” [15]. Specifically, clinicians expressed a desire for ABT education to be tailored to their setting, including the “when and how” [14,24]: “the need to tailor education and resources to a therapist’s specific non-SCI-specialized care settings was highlighted” [24] and “*I think maybe we just need more education on what [ABT equipment] we could use in our setting*” [14]. Clinicians also preferred accessible education, such as “*online modules”* [24], “*virtual learning”* [24], and “*social media*” [9] to assist with spreading ABT knowledge. Importantly, it was reported that different groups, not only clinicians, needed to increase their knowledge of ABT and “*change the attitude towards it … from the medical system, from the government, from the public, from everybody*” [9]. People with SCI not only “*made sure that [their] doctor knows about it as well”* but also informed their peers, “*recommend[ing] people getting into as soon as [they] can”* [10]. Participants also reported a need for “consensus on the definition and parameters of ABT” [11], recommendations for the appropriate use of technology [11,14], and the development of an evidence-based, standardized approach that considers the timing, intensity, and dosage of ABT [11].

### 3.4. Behavioural Change Wheel and COM-B Model

Evidence-based intervention functions that address the challenges in each category have also been listed (Table 3).

## 4. Discussion

According to our inductive thematic synthesis, there were many factors influencing the acceptance and adaptation of ABT across healthcare settings (Theme 1). Within this theme, we identified identity, knowledge, and health system as sub-themes. These challenges to implementing ABT may be addressed by Theme 2: proposed solutions. Motivation, empowerment, and advocacy were proposed to contribute to the initiation and sustained use of ABT. Beyond these factors, to help with implementation, participants also expressed a desire for standardized, evidence-based ABT educational content and training using different delivery methods.

### 4.1. The Behaviour Change Wheel, Intervention Functions, and Behaviour Change Techniques

According to our deductive analysis, all the components of the COM-B affected ABT implementation across healthcare settings in Canada [22]. However, three COM-B subcomponents were most limited according to themes and sub-themes from the inductive analysis. These subcomponents were social opportunity and physical opportunity, closely followed by reflective motivation. Consistent with the BCW, the intervention functions that aligned with both social opportunity and physical opportunity were restriction (e.g., increasing implementation of ABT by reducing the opportunity to engage in competing types of rehabilitation), environmental restructuring (i.e., altering the physical or social context), and enablement (i.e., increasing the means or reducing the barriers to increase capability or opportunity beyond education, training, and environmental restructuring). In the context of ABT in Canada, an example of environmental restructuring would be providing group functional electrical stimulation (FES) cycling sessions to reduce cost and staffing needs. Collaborative ABT goal setting between patients and clinicians would be an example of enablement. Modelling (e.g., participants in an ABT FES cycling session serve as examples for other patients) also matched with social opportunity, while training matched with physical opportunity (e.g., caregivers and patients take a training course to learn about ABT).

For the subcomponent reflective motivation, the suggested intervention functions were education (e.g., providing an information pamphlet about ABT), persuasion (i.e., using communication to stimulate action, positive or negative feelings about ABT), incentivization (i.e., creating presumption of a reward for engaging in ABT), and coercion (i.e., creating an expectation of punishment or cost for not engaging in ABT). There are several Canadian strategies in place that act as BCTs within these intervention functions outlined below; however, they do not necessarily span across the entire continuum of care. Therefore, we need to consider augmenting these strategies with additional BCTs.

### 4.2. Alignment of the Canadian ABT Community of Practice with Environmental Restructuring and Enablement Interventions to Target Social Opportunity

The ABT CoP acts as a practical social support and restructures the social environment around ABT (i.e., BCTs). The primary intervention functions that the ABT CoP support through their research, dissemination, and implementation strategies are environmental restructuring and enablement. These intervention functions target social opportunity as a source of behaviour and support the theme motivation, empowerment, and advocacy for ABT.

While the ABT CoP already includes two BCTs, the addition of ABT goal setting and restructuring the physical environment could reduce the challenges associated with social opportunity for ABT implementation. With a focus on discharge over neurorecovery in hospital settings, healthcare providers (HCPs) are faced with competing goals, yet individuals with SCI want ABT as early as possible [10]. Ørtenblad et al. also found that perspectives differed between outpatients with SCI and HCPs, who had to balance goal-setting criteria and practice [28,29]. Outpatients with SCI wanted to prioritize rehabilitation that would improve their quality of life; instead, HCPs prescribed assistive devices and used a compensatory approach. When implementing goal setting, it is important to formulate goals that are person-centred, which may be facilitated by ABT [29]. Goals should be set in collaboration with the patient according to the specific practice context. These goals should incorporate a flexible, pragmatic approach, spanning across transitions in care, instead of ending at the point of discharge from each specific setting [30].

To address challenges relative to the health system, the physical environment can be restructured (BCT) [22,23]. To facilitate smooth transitions in care, it is necessary to ensure that digital record systems are linked to each other within the same province or region [31]. Additionally, integrating the patient’s electronic medical record with a personalized integrated healthcare system could empower patients to advocate for continued ABT. Clinics outside of the traditional public system (e.g., community facilities or private ABT centres) may gain insight into patients’ ABT experience if access to their medical information is granted.

### 4.3. Alignment of Equipment and facilities with Training, Environmental Restructuring, Enablement, and Restriction to Target Physical Opportunity

To provide physical opportunity for ABT implementation, Gauthier et al. described equipment use for ABT programs across the continuum of care in Canada [8]. They noted high use of the low-tech equipment (e.g., parallel bars, fitness/training machines, splints/braces), while the medium- (e.g., elliptical, rower) to high-tech (e.g., exoskeleton, balance trainers) devices were used much less frequently. ABT equipment is part of physical environmental restructuring. A study by Renaud et al. showed that an emphasis on clinical knowledge and system management over advanced technology improved outcomes for SCI rehabilitation [32]. So, while using high-technology equipment may be important for one setting, ABT can also be achieved using low-technology equipment. This strategy can alleviate the cost of buying high-technology equipment combined with the cost of attending ABT sessions. However, the purchase of some high-technology devices includes training courses provided by the vendor to facilitate enablement.

In addition to training, action planning to improve enablement could further contribute to improved ABT implementation, including the use of equipment [23]. Action planning is the detailed planning of ABT implementation, in this case, and must include context, frequency, duration, and/or intensity [23]. According to Jesus and Silva, rehabilitation outcomes can improve through shared action planning between clinicians and patients [33]. This strategy should be implemented as early as possible and throughout the continuum of care.

Beyond hospital facilities in Canada, there are community centres and private gyms that provide ABT [9,34,35,36]. Not only do these places support the training of staff, but they also incorporate environmental restructuring, enablement, and restriction of conventional rehabilitation to focus on ABT and other strategies. However, in tackling challenges associated with transportation, travel, cost and staffing, telehealth could be considered to assist with enablement through restructuring the physical environment.

Telehealth removes the necessity for both HCPs and patients to travel, leading to cost savings [37]. By eliminating travel time, clinicians can attend to a greater number of patients within a given timeframe, addressing staff shortages. They can coach patients through ABT and monitor physiological functions (e.g., heart rate, respiration rate) through a web application [38]. However, the patient may need to cover the cost of support workers to assist with virtual therapy, depending on their level of function. Alternately, telehealth could function as a training tool to simultaneously instruct clinicians about ABT across various settings or watch recorded sessions. Telehealth also may allow clinicians to deliver ABT to patients with SCI living in rural or remote areas [37].

### 4.4. Alignment of Elective and Continuing Education Programs and Materials with Training, Education, Enablement, and Persuasion Interventions to Target Reflective Motivation

Both educational courses and materials provided by the ABT CoP align with a desire for education and training, according to our thematic synthesis. Education about and training for ABT is introduced as part of some Canadian university physical therapy and occupational therapy core courses, electives, or through continuing education (e.g., FES instructional courses [39]). In addition, these courses incorporate persuasion to engage in ABT, which improves reflective motivation.

Furthermore, educational materials have been created and disseminated by the ABT CoP [13]. Research activities such as peer-reviewed journal articles [8,9,15] and knowledge-sharing activities like ABT information postcards, a biennial ABT Expo, and Spinal Moves [13], a podcast about ABT, incorporate two BCTs. They provide information about health consequences (i.e., education) and represent credible sources (i.e., persuasion).

Incentivization through recognition and accreditation may improve the implementation of ABT. Specifically, these approaches represent incentive outcome and reward outcome BCTs. Continuing education for HCPs is effective and supports improvement in abilities, knowledge, approaches, and, ultimately, patient outcomes [40,41]. Accreditation helps to ensure that providers are credible and competent [42]. However, developing an accreditation process can be lengthy and time-consuming, involving numerous stakeholders (e.g., government, professional groups, accreditation board) [41,42]. With credible educational resources emerging and sponsored by groups such as Praxis Spinal Cord Institute and the Canadian ABT CoP, a first step towards accreditation might be determining learning objectives and organizing these materials into an online course. If clinicians, individuals living with SCI, their carers, or members of other groups complete course-related activities, they could be recognized with a certificate. A course executed in this manner could enable individuals to improve their knowledge about ABT delivery and increase the likelihood of ABT implementation.

### 4.5. Future Directions

Our review did not include the viewpoints of families or caregivers. The results were inclined towards the perspectives of some groups (e.g., clinicians, occupational therapists, physical therapists, individuals living with SCI) over others (e.g., clinic directors, administrators, advocates, policy makers). These findings reveal a gap in research activities, where future studies should investigate the perspectives of other key interest groups about ABT for SCI rehabilitation across the healthcare continuum in Canada.

This study illustrated the challenges to ABT implementation across the Canadian continuum of care and proposed solutions. Future research should aim to evaluate these suggested strategies and align them with appropriate policy changes. Policy changes can provide a reference for decision making and the allocation of resources towards ABT implementation [43].

## 5. Conclusions

In conclusion, to address the challenges of acceptance and adaptation of ABT across healthcare settings in Canada, we considered the proposed solutions from various key interest groups combined with the BCW framework. Specific BCTs should target restriction, environmental restructuring, enablement, modelling, training, education, persuasion, incentivization, and coercion. In turn, they will affect reflective motivation and physical and social opportunity, leading to the enhanced implementation of ABT.

## Figures and Tables

**Table 1:** Description of included articles exploring users’ perspectives on ABT.

Author(s)	Year	Methodology (Analysis Strategy)	Objective(s)	Inclusion Criteria	Healthcare Setting	Characteristics of Population	Sex/Gender	Number of Interviews or Focus Groups	Major Findings (e.g., Themes, Categories)	Key Takeaways
Cesca et al. [24]	2024	Qualitative exploratory (interpretive description)	To explore the knowledge, perspectives, and implementation of ABT among physical and occupational therapists in non-SCI-specialized centers.	Physical or occupational therapists working in a non-SCI-specialized center in Canada who have treated at least one person with SCI in the last 18 months.	Non-SCI-specialized centers (acute care, inpatient rehabilitation, long-term care, outpatient rehabilitation, rural outpatient clinic) across Canada (i.e., facilities lacking SCI-specific services).	7 participants; PTs (n = 4) and OTs (n = 3); Ontario (n = 4) and Alberta (n = 3); experience ranging from 3 to 22 years in current healthcare setting.	Not reported.	6 semi-structured interviews. All individual interviews, except one, completed with a PT and OT from the same facility.	Three themes: (1) available knowledge, resources, and therapy time in non-SCI-specialized centers challenge ABT implementation, (2) how current therapy practices in non-SCI-specialized centers align with ABT, and (3) desire for ABT knowledge.	The study emphasized the need for tailored ABT education in non-SCI-specialized centres.
Cheung et al. [9]	2022	Exploratory qualitative (interpretive description)	To understand how ABT was provided to Canadians with SCI/D in the community. To explore the use, perceived barriers, and facilitators of ABT and its associated technologies by therapists (e.g., PTs and OTs) and other clinicians.	Physical and occupational therapists, clinic employees, clinic managers, and clinic owners who work in a Canadian community-based center that offers ABT to individuals living with SCI.	Community-based ABT facilities across Canada.	13 participants; kinesiologists (n = 6), PTs (n = 4), OTs (n = 1), and clinic directors (n = 2). One PT was also a Clinician Scientist. Ontario (n = 5), Alberta (n = 3), Quebec (n = 1), and Saskatchewan (n = 1).	Male (n = 3) and female (n = 10).	10 interviews. Each interview consisted of one to two participants from a single site.	Overarching theme: ABT in the community is a client-centered approach characterized by various techniques, clinicians, and clientele. Three main categories within this theme: (1) characteristics of ABT in the community, (2) perceived challenges, and (3) need for advocacy.	The study revealed varied applications of ABT and related technologies across Canadian community-based facilities, unified by a focus on client goals and well-being. Systemic challenges hinder ABT’s implementation and accessibility in Canada. Solutions proposed include earlier ABT introduction, enhanced education, and cost reduction.
Coomaran et al. [25]	2022	Mixed methods, qualitative descriptive (conventional content analysis)	To characterize the overall experience of those involved in the community exercise program toward identification of key program elements.	Adults (i.e., ≥18 years of age) who (1) self-reported experience of chronic motor impairment related to a stable, neurological condition and (2) provided documentation of physician clearance for exercise.	Community exercise program delivered at the University of Regina Centre for Health, Wellness and Performance between September 2019 and March 2020.	11 individuals living with a neurological condition who participated in the community exercise program. Neurological conditions included incomplete SCI (n = 1), Parkinson’s disease (n = 1), mild TBI (n = 2), TBI (n = 1), ABI (n = 1), stroke (n = 3), and MS (n = 2). Time in program ranged from 4 weeks to 20 weeks.	Male (n = 4) and female (n = 7).	11 interviews with program participants. Interviews were conducted post-program activities in one-on-one format except in two cases where a spouse or caregiver supported the conversation. A sample of the program volunteers was also interviewed.	Four key program elements: 1. Support through supervision; 2. Social connection; 3. Individualized programming; 4. Experiential learning.	The program was feasible and effective in addressing the needs of older adults with varied levels and types of chronic neurological conditions. Key elements for success included personalized exercise prescriptions, social connections, and a supportive environment with knowledgeable supervision.
Jervis Rademeyer et al. [14]	2022	Exploratory, qualitative descriptive (interpretive description)	To determine if and how occupational and physical therapists in acute care hospital settings use ABT and its associated technologies.	Physical and occupational therapists licensed to practice in Canada and working in an acute care hospital setting with patients with SCI.	Acute care hospital settings in Canada.	7 participants from 6 sites spanning 4 Canadian provinces: PTs (n = 5) and OTs (n = 2).	Male (n = 1) and female (n = 6).	6 interviews. One interview with two participants from the same facility.	Three themes: (1) impact of patient acuity on ABT participation, (2) ABT approach unique to the acute care setting, and (3) influence of acute care work environment and therapy practice.	The study indicated that implementing ABT in acute care settings is difficult due to the high dosage of movement practice it demands. Enhancing ABT usage and dosage in these settings could be achieved through early patient education, leveraging social support, and integrating existing portable technology in acute care.
Jervis Rademeyer et al. [15]	2023	Qualitative (interpretive description)	To understand if and how physical and occupational therapists use ABT and its associated technologies for the rehabilitation of individuals living with SCI in inpatient and outpatient hospital settings in Canada.	Physical and occupational therapists licensed to practice in Canada and working at a rehabilitation hospital part of the Rick Hansen Spinal Cord Registry (RHSCIR).	Canadian rehabilitation hospitals participating in the RHSCIR.	22 participants from 9 rehabilitation sites across 8 Canadian provinces: PTs (n = 12) and OTs (n = 10).	Not reported.	10 focus groups, consisting of two or more participants.	Three overarching themes: (1) therapists’ decision-making approach to ABT and technology, (2) therapist perceived individual factors, and (3) access to ABT and equipment.	The application of technology in ABT varied, influenced by both tangible (e.g., equipment cost) and intangible barriers (e.g., departmental relations). ABT usage in Canadian rehabilitation hospitals is inconsistent. To enhance ABT utilization, ongoing education and development of tailored implementation strategies are recommended.
Kaiser et al. [8]	2023	Qualitative descriptive (conventional content analysis)	To understand and compare the perspectives of key interest groups on the challenges of implementing ABT in Canada for people living with SCI.	Canadian, English speaking, and either participated in, supervised, or had knowledge of ABT and SCI. A screening questionnaire was also used to query the nature and duration of experience with ABT to determine eligibility.	Canadian healthcare system.	48 participants representing 6 key interest groups: people with SCI (n = 10), hospital PTs and OTs (n = 6), community exercise trainers (n = 12), hospital and community administrators (n = 8), researchers (n = 7), and advocates, funders, and policy experts (n = 5). Experience/knowledge in ABT and SCI ranged from 0.25 to 33 years.	Male (n = 20) and female (n = 28). (M/F): people with SCI (7/3), hospital PTs and OTs (1/5), community exercise trainers (4/8), hospital and community administrators (1/7), researchers (4/3), and advocates, funders, and policy experts (3/2).	10 focus groups consisting of 2–6 participants. 2 one-on-one interviews.	Six themes: (1) challenge of gaps in knowledge and training, (2) challenge of standardizing ABT, (3) challenge of determining the optimal timing of ABT, (4) challenge of defining, characterizing, and achieving high dosage and intensity, (5) challenge of funding ABT, and (6) challenge of measuring participation and performance in ABT.	The study identified several challenges in implementing ABT in Canada, including gaps in knowledge and training, difficulties in defining and achieving appropriate dosage and intensity, funding challenges, and challenges in measuring participation and performance. These challenges varied among different interest groups, highlighting the need for tailored approaches to address these issues.
Singh et al. [26]	2018	Qualitative descriptive (conventional content analysis)	To understand how participation in Personalized Adaptive Locomotor Training (PALT) impacted participants’ lives, what aspects of PALT they perceived to work well, and what challenges they encountered while in the PALT program. To create recommendations, based on the identified challenges, to guide improvements to the design and implementation of PALT within Canadian tertiary SCI rehabilitation settings.	Traumatic, or non-progressive, non-traumatic, motor iSCI (AIS C or D), sub-acute stage of SCI (i.e., <one year post-injury), no deteriorating medical condition, capacity for generating lower extremity reciprocal alternating flexion/extension stepping pat- terns, compliance to reduce or eliminate the use of lower extremity orthotics, reside within 100 km of the training center, and access to reliable transportation.	Lyndhurst Center—TRI, part of the University Health Network in Canada (i.e., outpatient rehabilitation setting in Canada).	Traumatic SCI (n = 4), non-traumatic SCI (n = 3), AIS C (n = 1), and AIS D (n = 6). Neurological levels of injury ranged from C2 to T8.	Male (n = 5) and female (n = 2).	7 individual interviews conducted during the last week of the participant’s PALT.	Three main themes: (1) motives for participating, (2) perceived benefits, and (3) perceived challenges.	Participants reported significant physical and functional improvements from PALT but faced challenges in transferring skills learned in a controlled setting to daily walking. Specific challenges included neglect of other commitments, acquiring services for participation, re-integrating daily walking, and dealing with the rigid structure of PALT.
Singh et al. [27]	2018	Qualitative (thematic analysis)	To gain insight into participants’ perceptions of Personalized Adaptive Locomotor Training (PALT) and whether participation in PALT had an influence on their level of function and community living 1–2 years following discharge from PALT.	Previously participated in PALT and a semi-structured interview conducted upon completion of training, and to be able to participate in a telephone interview lasting 60 min.	PALT was conducted at the Lyndhurst Center—Toronto Rehabilitation Institute, part of the University Health Network in Canada (i.e., outpatient rehabilitation setting in Canada).	Traumatic SCI (n = 4), non-traumatic SCI (n = 2), AIS C (n = 1), and AIS D (n = 5). Neurological levels of injury ranged from C4 to T8. The age of the participants ranged from 49 to 65 years, and at the time of their interviews they had been living with SCI for between 1.9 and 2.7 years.	Male (n = 4) and female (n = 2).	6 individual interviews conducted 1–2 years following the participants participation in PALT.	Three main themes: (1) PALT outcomes, (2) continuing the rehabilitation journey, and (3) challenges experienced since discharge from PALT.	This study revealed that after discharge from PALT, individuals with SCI experience varying levels of physical and psychological adjustment. Most participants improved in psychological well-being after an initial decline. Challenges included difficulty adjusting, need for medical intervention, and importance of physical activity and social support. The study recommends routine follow-ups post-PALT for psychological well-being and emphasizes the need for socially supportive networks and less abrupt discharges.
Swaffield and Cheung et al. [10]	2022	Qualitative descriptive (conventional content analysis)	To capture the perspectives of individuals living with SCI on community ABT programs in Canada. To explore the benefits and challenges of ABT, the facilitators and barriers to accessing ABT in the community, and the motivations for participating in ABT programs.	Adults (i.e., ≥18 years of age) with chronic (>2 years post-injury) traumatic or non-progressive, non-traumatic SCI who participated in ABT within the past year for a minimum of two months at least, weekly in a community setting in Canada.	Canadian community healthcare setting.	Traumatic SCI (n = 10); involved in ABT programs in Ontario (n = 4), Saskatchewan (n = 4), Quebec (n = 1), and Nova Scotia (n = 1). Years of ABT ranged from 1 to 11.	Male (n = 6) and female (n = 4).	Ten individual interviews.	Overarching theme: ABT is a key part of their evolving and lifelong recovery process. Five categories within the theme: (1) motivation to initiate ABT, (2) participants’ experiences of recovery, (3) participants’ perceptions of how ABT contributes to recovery, (4) participants’ perceptions of factors limiting accessibility and participation in ABT, and (5) taking ABT to the next level.	ABT is viewed as crucial for continuous recovery and well-being in individuals with SCI. Enhancing awareness and accessibility of ABT could lead to increased participation in ABT programs.

ABT = activity-based therapy; SCI = spinal cord injury; PT = physical therapist; OT = occupational therapist; TBI = traumatic brain injury; ABI = acquired brain injury; MS = multiple sclerosis; AIS = American Spinal Injury Association Impairment Scale.

**Table 2:** Themes, sub-themes, and categories.

Theme	Sub-Theme	Category
1. Factors influencing acceptance and adaptation of ABT across healthcare settings in Canada	Identity	Person-specific considerations
		Professional roles and dynamics
		Goals of therapy
	Knowledge	Setting-specific considerations
		Current understanding of ABT definition and principles
	Health system	Cost
		Equipment, technology, and facilities
		Travel and transportation
		Time
		Staffing
		Transitions in care
2. Proposed solutions	Motivation, empowerment, and advocacy for ABT	
	Desire for education and training	

**Table 3:** Challenges identified through thematic synthesis mapped onto the Behavioural Change Wheel with associated intervention types proposed [22].

Category	Challenges Identified	Intervention Types Proposed
Physical capability—a person’s abilities arising from their physique and bodily functioning.		
Person-specific considerations	- Patient tolerance to therapy influences ABT approach; - Patient complexity disrupts opportunities for exposure to ABT; - Physical readiness to participate in intensive therapy early post-injury; - Unique personal challenges (e.g., hypotension, skin protection).	Training †; Enablement †.
Travel and transportation	- Concerns about distance to travel within the building.	
Psychological capability—a person’s ability to perform a behaviour arising from their psychological functioning.		
Person-specific considerations	- Mental readiness to participate in intensive therapy early post-injury; - Unique personal challenges (e.g., frustration).	Motivation, empowerment, and advocacy for ABT *: - Provide realistic hope/prognosis suggesting recovery potential to PLEX. Education †; Training †; Enablement †.
Transitions in Care	- Experiences of transition from rehabilitation hospital to community setting (e.g., decline in psychological well-being, disappointment, difficulty finding competent community PTs).	
Social opportunity—a person’s opportunity to enact a behaviour relating to the social world they inhabit, including the rules and norms that are operating and social cues.		
Professional roles and dynamics	- Professional role influences decisions made surrounding application of ABT; - Site-specific dynamics and teamwork influence structure of therapy program; - Reluctance to deviate from current routines and equipment; - Competition between departments (e.g., for grants, for equipment).	Motivation, empowerment, and advocacy for ABT *: - Advocate for ABT to the healthcare professionals with a focus on early use; - Increase support for ABT within the healthcare system. Restriction †; Environmental restructuring †; Modelling †; Enablement †.
Setting-specific considerations	- Focus on discharge over neurorecovery could lead to selection of compensatory over remediative approach in acute care and rehabilitation.	
Cost	- Need for and challenge of performing economic analyses to measure impact of ABT programs; - Desire for therapy being close to home.	
Staffing	- Value in social connection with other patients and student volunteers.	
Transitions in care	- Need for collaboration, continuity, and communication across continuum of care.	
Physical opportunity—a person’s opportunity to enact a behaviour that arises from objects and events in their environment, the space they inhabit, the time available, or the material and financial resources available to them.		
Person-specific considerations	- Need for medical clearance before engaging in ABT.	Training †; Restriction †; Environmental restructuring †; Enablement †.
Professional roles and dynamics	- Necessity of large team to deliver ABT to the optimal extent.	
Setting-specific considerations	- Limited vacancies in centres specializing in SCI care; - Increase in patients with non-traumatic SCI who are commonly referred to non-specialized SCI centres; - Medical acuity disrupts opportunities to engage in ABT in acute care; - Difficult to meet intensity requirements of ABT in acute care.	
Cost	- Challenge of funding is present across continuum of care; - Differences in public funding structures across provinces; - Expensive and often not covered by insurance or third parties; - Equipment can be costly to purchase, maintain, and/or use; - Cost of ABT to achieve desired intensity creates a financial challenge.	
Travel and transportation	- Challenge of commuting long distances; - Difficulty providing ABT to individuals in rural/remote areas.	
Staffing	- Staffing limitations impact ABT delivery; - Offering complementary therapies to alleviate staff demands.	
Equipment, technology, and facilities	- Difficulty accessing equipment and facilities (e.g., lack of floor space, delivering equipment to Canada, needing to share equipment); - Challenges of using technologies (e.g., finicky, cumbersome).	
Time	- Limited amount of treatment time due to heavy and unpredictable caseloads; - Equipment type, amount, and familiarity with can consume time; - Short length of stay disrupts opportunity to engage in ABT (e.g., “clean” patient); - Training for technology is time-consuming and intensive; - ABT was intense and time-consuming for PLEX but a worthwhile commitment.	
Transitions in care	- Readiness to participate often coincided with discharge from inpatient rehab.	
Reflective motivation—psychological processes of conscious planning and decision making.		
Person-specific considerations	- Therapists hesitant to engage in ABT when patient in mourning period.	Desire for education and training *: - Develop online ABT education; - Use social media to spread ABT knowledge; - Develop accessible information/education about ABT for patients and clinicians; - Ensure ABT education is tailored to setting; - Develop consensus on definition and parameters of ABT; - Develop recommendations on appropriate use of technology in ABT; - Have credentialing process in place for ABT training and education; - Develop evidence-based, standardized approach to ABT. Education †; Persuasion †; Incentivization †; Coercion †; Modelling †.
Goals of therapy	- Competing goals in therapy; - Challenges with tracking ABT progress (e.g., through outcome measures).	
Current understanding of ABT definition and principles	- Limited foundational understanding and application of specialized SCI knowledge and equipment; - Lack of knowledge and understanding about ABT across multiple key interest groups and settings; - Unsure of definition of ABT; - Knowledge of principles of ABT; - Lack of quality ABT education and training in hospital and community settings; - Lack of evidence on optimal timing, dosage, and intensity of ABT.	
Automatic motivation—involves (a) responding habitually or instinctively or (b) wants and needs arising from emotions or drives.		
Staffing	- Supervision of volunteers and staff made patients feel supported and motivated.	Motivation, empowerment, and advocacy for ABT *: - Prioritize patient motivation, an important factor to maintaining participation in ABT; - Promote high intensity to motivate PLEX to participate in program; - Incorporate educational components into program to empower patients; - Shift mainstream medical mindset to believe there is value in working below level of injury. Persuasion †; Incentivization †; Coercion †; Training †; Environmental restructuring †; Modelling †; Enablement †.
Current understanding of ABT definition and principles	- Performed ABT or components of ABT subconsciously in practice; - Interest in learning more about ABT to enhance skillset; - Clinician factors (e.g., education, school of thought) influence choice of ABT exercises and technology; - Advocacy needed for education (including ABT technologies and continuing education) for clinicians and clients.	

PLEX = people with lived experience; ABT = activity-based therapy; SCI = spinal cord injury; PT = physical therapist. * Sub-theme and categories developed from qualitative thematic synthesis. † Intervention function informed by Michie et al., 2011 [22].

## Data Availability

The data presented in this study are available on request from the corresponding author.

## Supplementary Materials

The following supporting information can be downloaded at https://www.mdpi.com/article/10.3390/healthcare12070703/s1, Table S1: The ENTREQ Checklist [17]; Table S2: Themes, sub-themes, categories, and additional supporting quotations. Table S3. Themes, sub-themes, categories, and additional supporting quotations.

## Appendix A. MEDLINE Search Strategy: PubMed Version

<1946 to 15 December 2023>

1. spinal cord injur*/
2. spinal cord injuries*/
3. spinal cord diseas*/
4. spinal cord injury/disease
5. paraplegi*/
6. quadraplegi*/
7. tetraplegi*/
8. Or/1–7
9. “activity-based therapy”
10. “activity based therapy”
11. ABT
12. “exercise therapy”
13. “locomotor therapy”
14. Or/9–13
15. qualitative/
16. perspectives/
17. Or/15–16
18. 8 and 14 and 17

## References

[1] World Health Organization Spinal Cord Injury 2013. https://www.who.int/news-room/fact-sheets/detail/spinal-cord-injury.

[2] Dromerick A.W., Lum P.S., Hidler J. (2006). Activity-based therapies. NeuroRx.

[3] Musselman K.E., Shah M., Zariffa J. (2018). Rehabilitation technologies and interventions for individuals with spinal cord injury: Translational potential of current trends. J. Neuroeng. Rehabil..

[4] Behrman A.L., Ardolino E.M., Harkema S.J. (2017). Activity-Based Therapy: From Basic Science to Clinical Application for Recovery After Spinal Cord Injury. J. Neurol. Phys. Ther..

[5] Karamian B.A., Siegel N., Nourie B., Serruya M.D., Heary R.F., Harrop J.S., Vaccaro A.R. (2022). The role of electrical stimulation for rehabilitation and regeneration after spinal cord injury. J. Orthop. Traumatol..

[6] Marquez-Chin C., Popovic M.R. (2020). Functional electrical stimulation therapy for restoration of motor function after spinal cord injury and stroke: A review. Biomed. Eng. Online.

[7] Zewdie E.T., Roy F.D., Yang J.F., Gorassini M.A. (2015). Facilitation of descending excitatory and spinal inhibitory networks from training of endurance and precision walking in participants with incomplete spinal cord injury. Prog. Brain Res..

[8] Gauthier C., Walden K., Jervis-Rademeyer H., Musselman K.E., Kaiser A., Wolfe D.L., Noonan V.K., Donkers S.J. (2023). Technology used in activity based therapy for individuals living with spinal cord injury across Canada. Spinal Cord. Ser. Cases.

[9] Cheung L., Musselman K.E., Kaiser A., Jervis Rademeyer H., Walden K., Marshall S., Gauthier C. (2022). Activity-based therapy in the community for individuals living with spinal cord injury or disease: Qualitative interviews with clinicians. Disabil. Rehabil..

[10] Swaffield E., Cheung L., Khalili A., Lund E., Boileau M., Chechlacz D., Musselman K.E., Gauthier C. (2022). Perspectives of people living with a spinal cord injury on activity-based therapy. Disabil. Rehabil..

[11] Kaiser A., Chan K., Sessford J., McCullum S., Athanasopoulos P., Rice C., Leo J., MacRitchie I., Zariffa J., Musselman K.E. (2023). Providing Insights into the Challenges of Implementing Activity-Based Therapy in Canada: A Comparative Analysis Using Focus Group Interviews with Key Interest Groups. Top. Spinal Cord. Inj. Rehabil..

[12] Musselman K.E., Walden K., Noonan V.K., Jervis-Rademeyer H., Thorogood N., Bouyer L., Chan B., Donkers S., Ho C., Jeji T. (2021). Development of priorities for a Canadian strategy to advance activity-based therapies after spinal cord injury. Spinal Cord..

[13] (2024). Canadian Activity-Based Therapy Community of Practice Vancouver, BC, Canada: Praxis Spinal Cord Institute. https://praxisinstitute.org/research-care/key-initiatives/activity-based-therapy/abt-cop/.

[14] Jervis Rademeyer H., Gastle N., Walden K., Lemay J.-F., Ho C., Marquez-Chin C., Musselman K.E. (2022). Activity-based therapy for individuals with spinal cord injury/disease: Perspectives of acute care therapists. Spinal Cord. Ser. Cases.

[15] Jervis Rademeyer H., Gauthier C., Zariffa J., Walden K., Jeji T., McCullum S., Musselman K.E. (2023). Using activity-based therapy for individuals with spinal cord injury or disease: Interviews with physical and occupational therapists in rehabilitation hospitals. J. Spinal Cord. Med..

[16] Thomas J., Harden A. (2008). Methods for the thematic synthesis of qualitative research in systematic reviews. BMC Med. Res. Methodol..

[17] Tong A., Flemming K., McInnes E., Oliver S., Craig J. (2012). Enhancing transparency in reporting the synthesis of qualitative research: ENTREQ. BMC Med. Res. Methodol..

[18] Kaiser A., Chan K., Pakosh M., McCullum S., Rice C., Zariffa J., Musselman K.E. (2022). A Scoping Review of the Characteristics of Activity-based Therapy Interventions Across the Continuum of Care for People Living With Spinal Cord Injury or Disease. Arch. Rehabil. Res. Clin. Transl..

[19] Behrman A.L., Harkema S.J. (2007). Physical rehabilitation as an agent for recovery after spinal cord injury. Phys. Med. Rehabil. Clin. N. Am..

[20] Hong Q.N., Pluye P., Fàbreques S., Bartlett G., Boardman F., Cargo M., Dagenais P., Gagnon M.-P., Griffiths F., Nicolau B. (2018). Mixed Methods Appraisal Tool (MMAT).

[21] Cheung L., McKay B., Chan K., Heffernan M.G., Pakosh M., Musselman K.E. (2023). Exploring sport participation in individuals with spinal cord injury: A qualitative thematic synthesis. J. Spinal Cord. Med..

[22] Michie S., van Stralen M.M., West R. (2011). The behaviour change wheel: A new method for characterising and designing behaviour change interventions. Implement. Sci..

[23] Michie S., Richardson M., Johnston M., Abraham C., Francis J., Hardeman W., Eccles M.P., Cane J., Wood C.E. (2013). The behavior change technique taxonomy (v1) of 93 hierarchically clustered techniques: Building an international consensus for the reporting of behavior change interventions. Ann. Behav. Med..

[24] Cesca N., Lin C., Abu-Jurji Z., Wexler A., Mark J., McCullum S., Kamran R., Chan B., Musselman K.E. (2024). Exploring knowledge and implementation gaps of activity-based therapy in centers lacking specialized spinal cord injury services: Understanding therapists’ perspectives. Spinal Cord. Ser. Cases.

[25] Coomaran V., Khan A., Tyson E., Bardutz H., Hopper T.D., Mang C.S. (2022). Evaluating and Characterizing an Individually-Tailored Community Exercise Program for Older Adults with Chronic Neurological Conditions: A Mixed-Methods Study. J. Aging Phys. Act..

[26] Singh H., Shah M., Flett H.M., Craven B.C., Verrier M.C., Musselman K.E. (2018). Perspectives of individuals with sub-acute spinal cord injury after personalized adapted locomotor training. Disabil. Rehabil..

[27] Singh H., Sam J., Verrier M.C., Flett H.M., Craven B.C., Musselman K.E. (2018). Life after personalized adaptive locomotor training: A qualitative follow-up study. Spinal Cord. Ser. Cases.

[28] Ortenblad L., Maribo T., Quistgaard B., Madsen E., Handberg C. (2023). The ambiguity of goal-setting: A study of patients’ perspectives on goal-setting in outpatient multidisciplinary rehabilitation of patients with spinal cord injury. Disabil. Rehabil..

[29] Ortenblad L., Maribo T., Quistgaard B., Madsen E., Handberg C. (2023). Goal-Setting in clinical practice: A study of health-care professionals’ perspectives in outpatient multidisciplinary rehabilitation of patients with spinal cord injury. Disabil. Rehabil..

[30] Maribo T., Jensen C.M., Madsen L.S., Handberg C. (2020). Experiences with and perspectives on goal setting in spinal cord injury rehabilitation: A systematic review of qualitative studies. Spinal Cord..

[31] Schofield P., Shaw T., Pascoe M. (2019). Toward Comprehensive Patient-Centric Care by Integrating Digital Health Technology with Direct Clinical Contact in Australia. J. Med. Internet Res..

[32] Renaud R., Locke H.N., Hariharan R., Chamberlain M.A., O’Connor R.J. (2018). Developing a Spinal Cord Injury Rehabilitation Service in Madagascar. J. Rehabil. Med..

[33] Jesus T.S., Silva I.L. (2016). Toward an evidence-based patient-provider communication in rehabilitation: Linking communication elements to better rehabilitation outcomes. Clin. Rehabil..

[34] (2023). First Steps Wellness Centre, Regina, SK: First Steps Wellness Centre. https://firststepswellnesscentre.ca.

[35] (2023). Taking Care of the Community with Your Help Since 2012: Walk It off Spinal Cord Recovery and Wellness Centre. https://walkitoffrecovery.org.

[36] ReYu (2023). Reconnect. Retrain. Redefine: ReYu Paralysis Recovery Centre. https://www.reyu.ca.

[37] Snoswell C.L., Taylor M.L., Comans T.A., Smith A.C., Gray L.C., Caffery L.J. (2020). Determining if Telehealth Can Reduce Health System Costs: Scoping Review. J. Med. Internet Res..

[38] Adams J., Lai B., Rimmer J., Powell D., Yarar-Fisher C., Oster R.A., Fisher G. (2022). Telehealth high-intensity interval exercise and cardiometabolic health in spinal cord injury. Trials.

[39] (2024). FES Courses: International Functional Electrical Stimulation Society, Inc. https://ifess.org/fes-courses-2/.

[40] Robertson M.K., Umble K.E., Cervero R.M. (2003). Impact studies in continuing education for health professions: Update. J. Contin. Educ. Health Prof..

[41] Travlos D.V., Baumgartner J.L., Rouse M., Wadelin J.W., Vlasses P.H. (2017). Forty Years of ACPE CPE Accreditation. Am. J. Pharm. Educ..

[42] Leist J.C. (2003). Accreditation: Standards for quality continuing professional development. J. Vet. Med. Educ..

[43] Canada Go (2021). Introduction to Policy: Canadian Heritage Information Network. https://www.canada.ca/en/heritage-information-network/services/digital-preservation/concepts-developing-policies/introduction-policy.html.

