# Supplementary material for: Implementing Activity-Based Therapy for Spinal Cord Injury Rehabilitation in Canada: Challenges and Proposed Solutions
Source: Healthcare (Basel). 2024 Mar 22;12(7):703. doi: 10.3390/healthcare12070703 (PMC11011823; doi:10.3390/healthcare12070703)
Supplement: Supplementary file 1 [file healthcare-12-00703-s001.zip › healthcare-2875177-supplementary.pdf]

Table S1. The ENTREQ Checklist (Tong *et al., 2012).*

**Item**	**Guide and description**	**Reported on page #**
Aim	State the research question the synthesis addresses.	Title and 2
Synthesis methodology	Identify the synthesis methodology or theoretical framework which underpins the synthesis and describe the rationale for choice of methodology (e.g., meta-ethnography, thematic synthesis, critical interpretive synthesis, grounded theory synthesis, realist synthesis, meta-aggregation, meta-study, framework synthesis).	2
Approach to searching	Indicate whether the search was pre-planned (comprehensive search strategies to seek all available studies) or iterative (to seek all available concepts until theoretical saturation is achieved).	2
Inclusion criteria	Specify the inclusion/exclusion criteria (e.g., in terms of population, language, year limits, type of publication, study type).	2-3
Data sources	Describe the information sources used (e.g., electronic databases (MEDLINE, EMBASE, CINAHL, psychINFO, Econlit), grey literature databases (digital thesis, policy reports), relevant organisational websites, experts, information specialists, generic web searches (Google Scholar), hand searching, reference lists) and when the searches were conducted; provide the rationale for using the data sources.	2

Electronic search strategy	Describe the literature search (e.g., provide electronic search strategies with population terms, clinical or health topic terms, experiential or social phenomena-related terms, filters for qualitative research and search limits).	Appendix A
Study screening methods	Describe the process of study screening and sifting (e.g., title, abstract, and full-text review, number of independent reviewers who screened studies).	2
Study characteristics	Present the characteristics of the included studies (e.g., year of publication, country, population, number of participants, data collection, methodology, analysis, research questions).	Table 1
Study selection results	Identify the number of studies screened and provide reasons for study exclusion (e.g., for comprehensive searching, provide numbers of studies screened and reasons for exclusion indicated in a figure/flowchart; for iterative searching describe reasons for study exclusion and inclusion based on modifications to the research question and/or contribution to theory development).	15 and Figure 1
Rationale for appraisal	Describe the rationale and approach used to appraise the included studies or selected findings (e.g., assessment of conduct (validity and robustness), assessment of reporting (transparency), assessment of content and utility of the findings).	12
Appraisal items	State the tools, frameworks, and criteria used to appraise the studies or selected findings (e.g. existing tools: CASP, QARI, COREQ, reviewer-developed tools; describe the domains assessed: research team, study design, data analysis and interpretations, reporting).	12 and Table 2

Appraisal process	Indicate whether the appraisal was conducted independently by more than one reviewer and if consensus was required.	12
Appraisal results	Present results of the quality assessment and indicate which articles, if any, were weighted/excluded based on the assessment and give the rationale.	Table 2
Data extraction	Indicate which sections of the primary studies were analysed and how were the data extracted from the primary studies (e.g., all text under the headings "results /conclusions" were extracted electronically and entered into a computer software).	3
Software	State the computer software used, if any.	Not Applicable
Number of reviewers	Identify who was involved in coding and analysis.	15
Coding	Describe the process for coding of data (e.g., line-by-line coding to search for concepts).	15
Study comparison	Describe how comparisons were made within and across studies (e.g., subsequent studies were coded into pre-existing concepts and new concepts were created when deemed necessary).	15
Derivation of themes	Explain whether the process of deriving the themes or constructs was inductive or deductive.	15
Quotations	Provide quotations from the primary studies to illustrate themes/constructs and identify whether the quotations were participant quotations or the author's interpretation.	16, 19-24, Table 3
Synthesis output	Present rich, compelling, and useful results that go beyond a summary of the primary studies (e.g., new interpretation, models of evidence, conceptual models, analytical framework, development of a new theory or construct).	16, 19-24, Tables 3 and S3

Table S2. Critical appraisal of included studies using the Mixed Methods Appraisal Tool (MMAT).

		Screening		Qualitative Studies					Mixed Methods Studies				
Author(s)	Year	S1	S2	1.1	1.2	1.3	1.4	1.5	5.1	5.2	5.3	5.4	5.5
Cesca *et al. [21]*	2024	Yes	Yes	Yes	Yes	Yes	Yes	Yes	—	—	—	—	—
Cheung *et al.* [9]	2022	Yes	Yes	Yes	Yes	Yes	Yes	Yes	—	—	—	—	—
Coomaran *et al. [22]*	2022	Yes	Yes	Yes	Yes	Yes	Yes	Yes	No (no rationale stated as to why mixed methods were chosen)	No (components of quantitative and qualitative studies not integrated)	No (components not integrated)	No (divergence and inconsistencies not discussed)	Yes
Jervis Rademeyer *et al.* [14]	2022	Yes	Yes	Cannot tell *(specific qualitative research approach not stated)*	Yes	Yes	Yes	Yes	—	—	—	—	—
Jervis Rademeyer *et al.* [15]	2023	Yes	Yes	Yes	Yes	Yes	Yes	Yes	—	—	—	—	—
Kaiser *et al.* [8]	2023	Yes	Yes	Yes	Yes	Yes	Yes	Yes	—	—	—	—	—
Singh *et al.* [23]	2018	Yes	Yes	Yes	Yes	Yes	Yes	Yes	—	—	—	—	—

Singh *et al. [24]*	2018	Yes	Yes	Cannot tell *(specific qualitative research approach not stated)*	Yes	Yes	Yes	Yes	—	—	—	—	—
Swaffield & Cheung *et al.* [10]	2022	Yes	Yes	Yes	Yes	Yes	Yes		—	—	—	—	—

Screening

S1. Are there clear research questions?

S2. Do the collected data allow us to address the research questions?

Qualitative Studies

1.1. Is the qualitative approach appropriate to answer the research question?

1.2. Are the qualitative data collection methods adequate to address the research question?

1.3. Are the findings adequately derived from the data?

1.4. Is the interpretation of results sufficiently substantiated by data?

1.5. Is there coherence between qualitative data sources, collection, analysis, and interpretation?

Mixed Methods Studies

5.1. Is there an adequate rationale for using a mixed methods design to address the research question?

5.2. Are the different components of the study effectively integrated to answer the research question?

5.3. Are the outputs of the integration of qualitative and quantitative components adequately interpreted?

5.4. Are divergences and inconsistencies between quantitative and qualitative results adequately addressed?

5.5. Do the different components of the study adhere to the quality criteria of each tradition of the methods involved?

Table S3. Themes, sub-themes, categories, and additional supporting quotations.

**Theme 1: Factors influencing acceptance and adaptation of ABT across healthcare settings in Canada**		
**Sub-Theme**	**Category**	**Quotations**
Identity	Person-specific considerations	*"So, if we think about post-injury, I find [the patient] might do 1 h of FES and they need a 2- hour break. [Therapists] would have to fill up their schedule based on what [the patient] can tolerate."* [15]<br><br>*"it's hard to just do so many repetitive things so consistently and not see progress for so long, like you really have to look down the long path to see progress."* [10]<br><br>*"I say to [physiotherapist] I'm not very helpful to you this last half hour because let me tell you I'm exhausted"* [22]
	Professional roles and dynamics	*"The benefits don't exceed the (set up) time (for technology) because our time limitation with people at the beginning when intensity of therapy is so important in that acute phase, in inpatient, I can't afford 10 min of a 45-minute session to get something set up and fiddle with it and get it going in just the right way. My hands can do that much faster by stimulating the muscle myself."* [15]<br><br>*"It's an interesting structure because we work as an OT and physio, but we're not an OT and physio department. So technically both the OT and physio are working for the same program which is the [name] program. So, when we're submitting a grant, we're automatically competing against each other because the grant is only going to be approved for one program."* [14]<br><br>*"We're very reliant on volunteers. Without volunteers, our FES program just wouldn't exist, I'll flat out say that. We need them to be able to run it the way we do."* [11]
	Goals of therapy	*"Especially in acute care they focus on discharging all patients that can be discharged first. Those are your priorities and then treatment comes after that."* [14]<br><br>"Clients' "*goals [were] so wide ranged*" (P01, 10.0years) and included walking, strengthening the limbs or core, improving hand or arm function, improving activities of daily living, and increasing independence." [9]<br><br>*"Common goals among participants focused on overall strength and endurance, balance, mobility, and participation in activities of daily living."* [21]<br><br>*"The tool we use I always criticize it for being not sensitive enough to pick up pretty good functional movement. So, one example of ours, like we've shown through research that 94% of our people have increased movement below their level of injury. One of the people who was in the 6% who didn't was able to ride a bike, like literally start it, stop it, but that's not a functional movement so you kind of feel like that person made huge progress, but it's not a functional movement as defined by a chart or by a developmental activity scale."* [11]
Knowledge	Setting-specific considerations	*"We do need them to tolerate up to an hour treatment without having hypertension, being dizzy, and having respiratory issues [because] our goal is to get them ready for rehab."*[14]<br><br>*"Even though we do have a longer length of stay, we often still have to balance how much time we can put towards activity-based therapy and function compared to discharge planning and other goals."* [15]

	Current understanding of ABT definition and principles	*"I would say for the most part ... I'm doing [ABT] kind of unconsciously … there would be a goal of neuro recovery [in therapy]."* [21]<br><br>*"We are not still clear in many of the sites what ABT is, so we are still struggling with how this is and what is conventional therapy."* [11]<br><br>*"the medical community uh sees no value in referring to community-based therapy. So, if there is a client that is, you know a complete injury, well they don't think there is additional value to continue to work below the level of injury."* [9]<br><br>*"I think the biggest and one of the barriers is people not knowing, so they don't know what they're kind of capable of because they're not put into different um like into that environment."* [10]<br><br>*"You need to be able to measure how intense the workout is and how hard the person's working. So, I think cardiovascular measures correlated to distances and repetitions and weights...understanding is that heart rate elevating? Are they breathing heavily? Are they actually working hard? How much effort are they putting in? And then, what are they actually achieving with that effort? How many reps are they doing? How far are they going? How much weight are they lifting? How many steps are they taking? Whatever those measurements are to be able to paint that picture of how hard somebody's working and how that correlates to their improvement in function."* [11]
Health System	Cost	*"we don't want a piece of equipment, the ratio of something costing $200,000, and only 20% of our population being able to use it."* [9]<br><br>*"I was injured while I was working so I do have [workplace insurance] and they cover the cost […] if I didn't have insurance, I probably wouldn't be able to go."* [10]<br><br>*"Us being a charity...we've been able to get a lot of our equipment donated. So, while a lot of people don't want to fund therapy costs for individual clients, I think we have about $500,000 worth of equipment in our facility that's all been bought through donations to (facility) for those specific pieces. So, I think that's helped a lot, being able to get a lot of that equipment."* [11]<br><br>*"This is a systemic problem. I think our length of stays are shortening, our outpatient therapies are shrinking, there's no home care therapy without paying for it out of pocket, over 80% of people who have spinal cord injuries in this province do not have means of third-party funding to afford therapies if they're out of their own pocket, and that there is no clear systemic process to achieve long-term ABT the way the system presents itself today."* [11]
	Equipment, technology, and facilities	*"Spinal cord injury is a relatively equipment-heavy diagnosis … it hasn't always been the priority for us to have that stuff, so we're often borrowing ... equipment."* [21]<br><br>*"The first barrier to think about is just process. So, if we were going to implement a new approach or a new therapy, we would just need to make sure we have a good change management process, like how it's going to change the way and the journey of the patient in their rehab stay here. So that would be more of a process issue to look at what are we currently doing and how do we integrate this in a meaningful way. Financially and sort of structurally; if we're looking at putting any additional space, equipment needs, you just have to really look at the space that you have, what*

		could go into it as it is or what would need to be modified if you needed more square footage." [11]
	Travel and transportation	*"sometimes just getting ready for the transportation, like the uh care that they need and how much it takes for them to get out of their house depending on their level[…] if their caregivers don't show up, they can't come in cause they can't get out of bed."* [9]<br><br>"Currently, this clinic has clients *"from Whitehorse all the way down to you know, the United States."" [10]*
	Time	*"each trainer gets an observation, a supervised period, and a hands-on period...so that helps us get to know the equipment and the technology."* [9]<br><br>*"I always think of [my large] caseload and just like how much time we actually have to dedicate to each person."* [21]
	Staffing	*"There is a physical educator. The physical educator always does a pre-test to detect the physical condition. After that, he will adapt a training program and he will call us in physio to verify the upper limbs, if there are any particular contraindications." [15]*<br><br>*"it's easier to get insurance companies to pay for physio."* [9]
	Transitions in care	*"I only see acute care rehab patients. Even if they come back into acute care, but they don't have a spinal cord need...it's considered a medicine-related issue. They don't come to me, and I can't follow them either. They're not my patient."* [14]<br><br>*"Right after it ended I was pumped, I wanted to walk. I was going to walk myself but after a while you realize you need still a professional somebody who will...tell you are doing it right or you were doing it wrong so that was missing... I can walk on a walker and I feel happy with that but when I came home reality hit me really hard." [23]*
**Theme 2: Proposed solutions**		
**Sub-Theme**	**Category**	**Quotations**
Motivation, empowerment, and advocacy for ABT		*"I feel like if it could be introduced earlier, like during your inpatient time, I feel like that's what I would want right now to change."* [11]<br><br>*"'you broke your neck, you're never gonna walk again' […] hearing words like that can really have a negative impact on them and their recovery and also their quality of life going forward and how they really adjust and cope with their injury."* [10]
Desire for education and training		*"Yeah, I wouldn't mind [learning more about ABT]. I feel like I'm doing [ABT] but like what else could I be doing."* [21]<br><br>*"That's one thing we've always found, is clinicians working in acute care, there is very little adapted type of training for people who work in acute care, in anything in physiotherapy, it's mostly always external or rehab." [14]*<br><br>*"To recommend what will work, what will not work, I don't think we have enough evidence to say...Just for neuromodulation, there are so many different kinds of neuromod machines itself, so we don't know what we're using will work with what, so lot of unknowns out there."* [11]

ABT = activity-based therapy; FES = functional electrical stimulation; OT = occupational therapist.
